# Editorial: Lymphatic delivery and targeting of drugs, vaccines, and imaging agents

**DOI:** 10.3389/fphar.2022.1011778

**Published:** 2022-09-02

**Authors:** Sifei Han, Natalie Trevaskis, Pavel Gershkovich, Leonid Kagan

**Affiliations:** ^1^ Drug Delivery, Disposition and Dynamics, Monash Institute of Pharmaceutical Sciences, Monash University, Parkville, VIC, Australia; ^2^ School of Pharmacy, Biodiscovery Institute (BDI), University of Nottingham, University Park, Nottingham, United Kingdom; ^3^ Department of Pharmaceutics and Center of Excellence for Pharmaceutical Translational Research and Education, Ernest Mario School of Pharmacy, Rutgers, The State University of New Jersey, New Brunswick, NJ, United States

**Keywords:** Drug delivery, lymphatics, imaging, macromolecules, nanomedicine, immunotherapy

The lymphatic system plays a key role in the (patho) physiology of a number of diseases, including autoimmune/inflammatory disorders, cancer, infections, and metabolic syndrome. Biological pathways in the lymphatic system provide new promising treatment targets for several conditions (Oliver, Kipnis et al., 2020; Petrova and Koh 2020). However, most molecules, including imaging and therapeutic agents, exhibit poor access to the lymphatics which impedes research of the lymphatic system and development of related therapies (Trevaskis, Kaminskas et al., 2015). In this Research Topic, we present a collection of primary research articles that provide new insights into the physiology of lymphatics and lymph-directed drug delivery, as well as review articles that summarize the approaches used to improve lymphatic targeting of imaging agents and drugs.

The lymphatic vessels are the conduit for the transport of lymph fluid from the periphery to lymph nodes and eventually to the systemic blood circulation. This function is enabled by a specific lymphatic vasculature network, starting from fine initial capillaries to collecting lymphatics vessels and all the way to major lymph ducts. Mapping the branching of the lymphatics is critical for better understanding of the flow dynamics and functions of the lymphatic system. Talkington et al. (Talkington, Davis et al., 2022) studied the skin lymphatics in mice and found that the branching profiles of lymphatic vessels follow a unique pattern that is different from typical Murray’s law (Murray 1926). Murray’s law is a branching rule that is applied in diverse systems, including leaf venation, airways, veins and arteries. However, in the case of the skin lymphatic vessels, the authors found that the daughter vessels are smaller relative to the parent vessels (with a Murray’s Law exponent of approximately 1.45) than would be predicted by typical Murray’s Law (with an exponent of 3). The authors hypothesize that the lymphatic branching structure may be optimized to enhance lymph mixing, particle exchange, or immune cell transport. The findings deliver new insights into the physiology of the lymphatics and provide a useful reference for researchers in the fields of immunology, vaccinology and drug delivery.

The above findings would not have been possible without advanced imaging methodologies, as the lymphatics are difficult to locate and observe. Due to the small, fragile and one-way flow feature, it is also difficult to inject imaging agents directly into the lymphatics. New delivery approaches are therefore needed to facilitate access of imaging agents to the lymphatics. A comprehensive review by Russell et al. summarizes contemporary knowledge regarding fluorescent tracers for enhanced lymphatic imaging. The authors briefly highlight the physiology of the interstitium (where lymphatic capillaries begin), followed by the general principles of (macro)molecule transport via the lymphatics. Subsequently, various types of fluorescent imaging agents are introduced in line with their lymph-targeting mechanisms. Fluorophores with distinct excitation/emission patterns may be associated with synthesized nano-sized particles or endogenous biological carriers (proteins, cells, etc) to promote access to the lymphatics. A range of characteristics, including size, shape, charge, weight, conjugates stability, and quantum yield collectively impact lymphatic targeting and imaging quality of fluorescent agents. The review article provides important knowledge and a handy toolkit for medical researchers and diagnostic product development.

In addition to targeting of fluorescent tracers for imaging purposes, drug delivery to the lymphatic system has captured more and more attention from medical scientists due to the growing realisation of the potential therapeutic opportunities provided by targeting this system (Trevaskis, Kaminskas et al., 2015). In the current collection, McCright et al. review nanoparticles for lymph-directed delivery including polymer-, liposome/micelle-, dendrimer-, and solid lipid particle-based systems. Nanoparticles can be engineered to achieve delivery to specific regions within the lymphatic system including distinct regions within the lymph nodes, certain immune cell populations, or the lymphatic endothelium. In addition to synthetic drug carriers, the author also introduced how endogenous lymphotropic macromolecules, such as lipoproteins, may be exploited for lymphatic drug delivery. The article provides a timely overview of the principles and approaches for lymphatic targeting of therapeutic agents.

Delivery of therapeutic agents to the lung lymphatics may provide opportunities for improved treatment of respiratory system-related diseases. However, this is more challenging compared to delivery to the lymphatics in other tissues, such as skin and muscle, where subcutaneous or intramuscular injection can be utilized to penetrate the skin barrier to access the interstitium (thus initial lymphatic capillaries). In contrast, permeation of macromolecules across mucosal barrier in the lung is difficult and highly variable. This may at least partially explain why inhaled macromolecular drug delivery systems often demonstrate conflicting results with regards to drug access to the lung lymphatics. Ibrahim et al. evaluated lymphatic uptake of radio-labelled model liposomes following administration via nebulization to sheep cannulated at the caudal mediastinal lymph duct to collect the lung-draining lymph. In this study, there was very limited uptake of liposomes into the lung lymphatics. This quantitative *in vivo* study provides an important reference for researchers interested in delivery to lung lymphatics.

In addition to providing potential benefit through enhanced delivery to pharmacological targets in the lymphatic system, lymphatic drug transport also offers a route for orally administered drugs to avoid hepatic first-pass metabolism and therefore enhance systemic bioavailability (Shackleford, Faassen et al., 2003). Hepatic first-pass metabolism, which occurs during drug passage through the liver following absorption from the intestine into the portal vein, can cause low and variable drug bioavailability, and thus challenge drug efficacy and safety profiles. The studies by Quach et al. in the current collection demonstrate the application of a triglyceride prodrug template to direct drugs to the lymph via integration into dietary lipid transport pathways, thereby avoiding passage via the portal vein to the liver and hepatic first pass-metabolism. This approach is shown to enhance the oral bioavailability of buprenorphine, an opioid analgesic with substantial first-pass metabolism, by 22-fold. The lymph-directing glyceride prodrug approach has potential to enable the development of an oral buprenorphine product, which may be preferred by patients and caregivers compared to current parenteral and sublingual formulations.

In summary, the publications in this Topic highlight the important applications of lymphatic delivery of drugs and imaging agents for both fundamental research and therapeutic development. They summarize tools and approaches that may be used to enhance lymphatic delivery, and remind the research community of the urgent need for more efficient and effective lymphatic delivery strategies to provide therapeutic solutions for the expanding number of recognized diseases involving the lymphatic system.
